# Experiences and perceptions on diagnostic delay of leprosy by affected people in Colombia: A qualitative study

**DOI:** 10.1371/journal.pntd.0014412

**Published:** 2026-06-11

**Authors:** Heleen Neeltje Willemijn Duighuisen, Daniel Gonzalo Eslava Albarracin, Anil Fastenau, Nimer Ortuño-Gutiérrez, Srilekha Penna, Alena Kamenshchikova

**Affiliations:** 1 Department of Health, Ethics and Society, Faculty of Health, Medicine and Life Sciences, Maastricht University, Maastricht, The Netherlands; 2 German Leprosy and Tuberculosis Relief Association (GLRA/DAHW), Bogotá, Colombia; 3 Escuela de Enfermería, Fundación Universitaria Cafam, UNICAFAM, Bogotá, Colombia; 4 Department of Global Health, Institute of Public Health and Nursing Research, University of Bremen, Bremen, Germany; 5 German Leprosy and Tuberculosis Relief Association (GLRA/DAHW), HQ, Wuerzburg, Germany; 6 Heidelberg Institute of Global Health, University of Heidelberg, Heidelberg, Germany; 7 Marie Adelaide Leprosy Centre, Karachi, Pakistan; 8 Program Department, Damien Foundation, Brussels, Belgium; 9 German Leprosy and Tuberculosis Relief Association (GLRA/DAHW), New Delhi, India; 10 Department of Social Medicine, Maastricht University, Maastricht, The Netherlands; 11 Department of Medical Microbiology, Infectious Diseases and Infection Prevention, Maastricht University, Maastricht, The Netherlands; Adolfo Lutz Institute: Instituto Adolfo Lutz, BRAZIL

## Abstract

Leprosy, also known as Hansen’s disease, is an infectious neglected tropical disease that requires a timely diagnosis and onset of multi-drug therapy to cure, halt transmission and prevent irreversible disabilities. Although Colombia shows a relatively low incidence of leprosy, the disease remains endemic in certain parts of the country. In addition, high rates of leprosy-related disability are observed due to diagnostic delay. Patients’ experiential knowledge and expertise are crucial to further understand the reasoning behind this delay in diagnosis. Therefore, our study aimed to explore the experiences and perceptions of people affected by leprosy with diagnostic delays in the departments Cesar and Valle del Cauca in Colombia where the disease is endemic. We conducted 24 semi-structured in-depth interviews with people affected by leprosy in Colombia and used thematic analysis to analyse the interview results. Based on our analysis, we mapped out the patient pathway towards diagnosis and treatment, and highlighted the individual-level, community-level, and health system-level challenges leading to potential delays. Main reasons for delay included perceived limited leprosy training and expertise among healthcare workers, challenges related to the organisation of leprosy care, accessibility and affordability barriers, limited awareness about leprosy and stigmatisation of people diagnosed with leprosy. A multifactorial approach to leprosy diagnostic delay is necessary to tackle current challenges, including investing in integral leprosy care centres and prioritising active case detection. Implementing leprosy awareness strategies among healthcare workers and communities are necessary to tackle the stigmatisation of the disease and improve overall leprosy knowledge and expertise.

## Author summary

This study addresses the problem of diagnostic delay of leprosy in Colombia. Despite a relatively low leprosy incidence in Colombia, leprosy continues to be endemic in certain regions, and the country shows high rates of disability at the time of diagnosis due to diagnostic delay. Through qualitative analysis, 24 interviews with people affected by leprosy were conducted to explore the current barriers to timely diagnosis and treatment following their experiences and perceptions. Our findings highlight five major reasons leading to diagnostic delay along the patient pathway towards diagnosis and treatment, including limited training and experience with leprosy among healthcare workers, stigmatisation of people diagnosed with leprosy, challenges related to the organisation of leprosy care, accessibility and affordability barriers, and limited awareness and knowledge about leprosy among the general population. The study concludes that diagnostic delay is complex and often an accumulation of multiple challenges on the individual-level, community-level, and health system-level, therefore requiring a multifactorial approach to lower diagnostic delays in our study setting. Recommendations for systemic improvements include prioritising active case detection, contact tracing activities and improving medical expertise among healthcare workers. Investing in integral leprosy care centres and implementing awareness strategies among healthcare workers and the general population are essential to overcome current barriers, as well as overall commitment by government agencies, healthcare policymakers and healthcare workers.

## Introduction

Leprosy, also known as Hansen’s disease, is an infectious neglected tropical disease caused by *Mycobacterium Leprae,* predominantly affecting the skin and peripheral nervous system. A timely diagnosis and onset of multi-drug therapy (MDT) are crucial to cure the disease, halt transmission, prevent irreversible disabilities and eliminate leprosy [1]. Nevertheless, diagnostic delay remains a problem, contributing to various degrees of disability in patients at the time of diagnosis [2]. Hence, the World Health Organisation (WHO) highlights diagnostic delay as a major challenge in its Global Leprosy Strategy 2021–2030 and aims for a 90 per cent global reduction of new Grade 2 Disability (G2D) cases by 2030 [2]. Various causes for diagnostic delay have been mentioned in previous research across the globe. For instance, according to a global systematic review from 2021 by Dharmawan and colleagues, lack of awareness about leprosy symptoms, rural residence and stigma are among the individual and community factors that can be seen contributing to delay in leprosy diagnosis [3]. Healthcare-related factors involve poor geographical access to healthcare services, limited availability and low commitment of healthcare workers, as well as a lack of integrated leprosy services [4].

In Colombia, there is a relatively low incidence of leprosy with approximately 200–300 new registered cases annually [5]. Despite not being considered an official public health problem due to national prevalence rates lower than one per 10,000 population, leprosy remains endemic in certain parts of the country, including the departments of Cesar and Valle del Cauca [5]. In 2022, 19 new leprosy cases were registered in Cesar and 38 in Valle del Cauca (including its district Cali). New case detection rates were 1.24, 1.43 and 0.74 (per 100,000 population) in 2023 for respectively Cesar, Valle del Cauca and its district Cali, while the national average was 0.49 [5]. Simultaneously, the country shows high rates of disability at the time of diagnosis, which might be an indicator of diagnostic delay. While 63 per cent of the new cases in Cesar had some degree of disability at the time of diagnosis, this was 29 per cent in Valle del Cauca (compared to a national average of 39 per cent) [6]. Two previous studies from 2013 and 2020 highlighted average diagnostic delay times of approximately three years in Colombia [7,8]. More recent research from 2023 by Rodríguez Torres and colleagues showed an average delay time of 21.5 months in the Valle del Cauca department [9]. The impact of diagnostic delay on Colombian leprosy patients has been described by Barbosa Ladino and colleagues who highlighted both negative physical and psychological consequences, such as the worsening of physical symptoms and experiencing fear and discrimination due to experiences of not being taken seriously by healthcare workers [10]. To start unpacking the reasoning behind the delay of leprosy diagnosis in Colombia, our research group has previously conducted a qualitative study among leprosy health professionals in Cesar and Valle del Cauca to explore their perspectives on diagnostic delay [11]. We illustrated the complexity of delay by mapping out how it builds on various factors on individual-, community- and health system-levels [11].

Previous research focused on the perspectives of leprosy health professionals on diagnostic delay in Colombia or on settings outside Colombia. There is still limited understanding of the lived experiences and perceptions by people affected by leprosy with diagnostic delay in Colombia. Engaging with patients as experts is a valuable research approach and aligns with studies in different areas of healthcare about diagnostic delay, such as tuberculosis and cancer [12–14]. As explained by Staley and colleagues, patients’ experiential knowledge is a crucial source of knowledge, specifically to generate potential solutions for early leprosy diagnosis [15]. Therefore, building on the previous research, this study aims to explore the diagnostic pathways, experiences and perceptions on diagnostic delay by people affected by leprosy in the Cesar and Valle del Cauca departments in Colombia.

## Methods

### Ethics statement

This study was approved by the Ethics Committee of the Faculty of Health Sciences, the Francisco de Paula Santander University, Colombia (ethical clearance registration number: CEI-ISEM-03–2023: ENFERMERÍA) and the Global Health Ethics Committee of the Faculty of Health, Medicine and Life Sciences, Maastricht University, the Netherlands (ethical clearance registration number: FHML/GH_2023.005). We followed the guidelines of the British Sociological Association concerning professional integrity, participant relationships, data storage and publication practices [16]. Informed written consent was obtained from the participants before data collection.

### Study setting

This research focused on the two regions Cesar and Valle del Cauca in Colombia where leprosy is endemic. The departments were chosen as research setting due to the availability of connections necessary for sampling procedures and the varying characteristics of both departments, including geographical location, population density and disability grade. Cesar is located in Northern Colombia and is divided into 24 municipalities. With a population density of approximately 61 inhabitants per square kilometre, it encompasses relatively more rural areas than Valle del Cauca [17]. Valle del Cauca is situated in Western Colombia, divided into 42 municipalities and has a population density of approximately 209 inhabitants per square kilometre.

In Colombia, health services are organised under a universal insurance-based system with mandatory enrolment, ensuring access to medical care through either the contributory or subsidised schemes. The Colombian National Strategic Plan 2016–2025 aims to guide towards the elimination of leprosy by the end of 2025 [18]. Its three pillars focus on strengthening governance, improving the quality and efficiency of leprosy-health services, and eliminating discrimination caused by the disease. Specific attention is paid to lowering the number of G2D cases, by aiming to diminish this rate to 0.46 cases per million by 2025 [18]. The Ministry of Health and Social Protection and the National Institute of Health direct the National Leprosy Program, including planning prevention, surveillance, and control activities at national level [18,19]. Implementation of leprosy control activities is decentralised to local health directorates on departmental/district and municipal level [18,19]. However, multiple barriers have been described that impede successful implementation, such as limited financial and human resources and poor hospital infrastructure [20].

Leprosy care is provided free of charge, regardless of insurance status, and is integrated into both public and contracted private healthcare providers. Diagnosis, MDT, and clinical follow-up are financed by the government and are primarily delivered at primary care facilities, with referrals to specialised centres when needed. Although patients are not expected to encounter direct costs, a small number of patients may seek private services or face out-of-pocket expenses when confronted with administrative barriers, delays in service authorisations, difficulties accessing specialised providers, or geographic limitations, particularly in rural and remote areas [21,22]. Both the WHO and national guidelines consider clinical examination as the most important for diagnosis, possibly complemented with additional tests (i.e., slit skin smear test or biopsy) [19,23].

### Study design and study population

We conducted a qualitative investigation using semi-structured in-depth interviews among people affected by leprosy. Diagnostic delay was defined as the time between the onset of the first leprosy symptoms and the moment of conclusive leprosy diagnosis by a healthcare worker. By engaging with the lived experiences of people affected by leprosy, we scrutinised the diagnostic journey towards the diagnosis and aspired to uncover potential elements causing delays, including the underlying social, moral and political assumptions that might contribute to delay.

The study population consisted of persons previously diagnosed with leprosy and living in the Cesar or Valle del Cauca department. Inclusion criteria consisted of the following: adults aged above eighteen years, being diagnosed with leprosy, and residing in the Cesar and/or Valle del Cauca department during the period of symptom onset until diagnosis. Due to the relatively low number of leprosy cases, it was decided not to set any restrictions on the year of diagnosis nor the disability grade at the moment of diagnosis. Convenience sampling was used to recruit participants through the network of the German Leprosy and Tuberculosis Relief Association (GLRA/DAHW) Colombia, which works together with the Colombian Health Secretariat. During regular monthly patient meetings hosted by GLRA/DAHW, the study was introduced to the attending people affected by leprosy and all attendees were invited. If expressed interest in the study, we provided potential participants with detailed information and informed consent forms. Approximately 35–40 people affected by leprosy were initially invited to participate during the monthly meetings, after which 24 agreed to take part. None of the 24 participants who consented to participate dropped out of the study.

### Data collection and analysis

A total of 24 interviews were conducted in July and August 2023 by an experienced local research member of GLRA/DAHW (DGEA), who shares a similar cultural and linguistic background with the participants. The number of interviews was determined based on the research objectives and the availability of participants within the leprosy patient population. The first interviews were used as pilot interviews; it was not necessary to make any adjustments to the interview guide. At the 15^th^ interview, the information shared by the participants was repeated and no new information emerged, therefore we stopped the recruitment process. However, to ensure data saturation, we continued with already scheduled interviews that allowed us to confirm saturation.

A summary of participant demographics can be found in Table 1. Participants were invited to attend one of the four interview days, two for each department. Interviews were held at public spaces, such as a hotel lobby, to embrace an informal atmosphere while simultaneously ensuring a calm environment. Participants were free to bring anyone to make them feel more comfortable. Interviews took place in Spanish and were recorded with an external recording device. Interviews lasted 24:15 minutes on average, ranging between 12:27 and 35:59 minutes. A semi-structured interview guide with mainly open-ended questions was followed to encourage participants to elaborate on their answers. The interview guide consisted of the three broad themes, including patient’s pathway, their perception of diagnostic delay and patient’s suggestions for diminishing the delay (S1 Appendix). We developed an interview guide following the experiences from the previous research on diagnostic delay in Colombia [7,11].

**Table 1:** Participant demographics.

Variables		Number of participants (total = 24)
Sex	Male	9
	Female	15
Age	70-76	6
	60-69	1
	50-59	8
	40-49	3
	30-39	4
	23-29	1
	unknown	1
Department	Cesar	12
	Valle del Cauca	12
Disability Grade	G2D	11
(at diagnosis)	G1D	6
	G0D	7
Year of diagnosis	2014-2023	16
	2004-2013	2
	1994-2003	4
	1988-1993	2

All interviews were transcribed verbatim into Microsoft Word documents. The method of thematic analysis was applied following Braun and Clarke’s approach using Microsoft Excel by the first author HD [24]. A combination of inductive and deductive strategies was used for thematic analysis. This allowed us to follow the pre-determined themes while exploring possible additional topics. As a quality assurance measure, the coding tree and subsequent themes were discussed and agreed upon by HD, DGEA and AK. A total of 31 codes were generated and grouped into the three pre-determined themes. No new themes were generated during the analysis. However, it was decided to divide the theme ‘patient pathway’ into sub-themes to indicate the various steps along the patient trajectory towards diagnosis. The final analysis of the interview data was written by HD and shared with the remaining authors for review. No disagreements were recorded throughout the analysis process. After we completed the thematic analysis, the various reasons for delay along the patient pathway were distinguished between individual-level, community-level, and health system-level challenges, which is reflected in the results section. The quotes used in the results section were translated to English.

## Results

We started the interviews by asking participants to outline their route towards diagnosis, including the initial symptoms and the moment of diagnosis. Based on the interviews, we were able to create an estimated timeline of delay until diagnosis for our participants, which varied between no delay and 20 years (Table 2). However, it has to be noted that the shown delay times represent estimates based on the participants’ recollections of their diagnostic journeys, therefore they may reflect a potential recall bias. Four participants were diagnosed within six months after symptom onset. Important to note that three of them indicated that they had a family member who was diagnosed with leprosy or who was going through the process of being diagnosed, which may have been a contributing reason for their faster diagnosis. For thirteen participants, the delay time lasted at least two years. Three participants experienced delays between 10 and 20 years; these participants were diagnosed between 2018 and 2023 and resided in the department of Cesar. Although the interviews focused primarily on the delay time until diagnosis, it appeared that experiencing delay was not limited to the moment of diagnosis only, as it was also observed in the periods between diagnosis and the start of treatment.

**Table 2:** Estimated diagnostic delay according to the constructed patient pathway.

Delay in time (first symptoms until diagnosis)	Number of participants (total = 24)
≥10 ≤ 20 years	3
≥5 < 10 years	4
≥2 < 5 years	6
≥1 < 2 years	4
≥6m < 1 year	3
<6 months	4

### Reasons for delay

**Fig 1:**
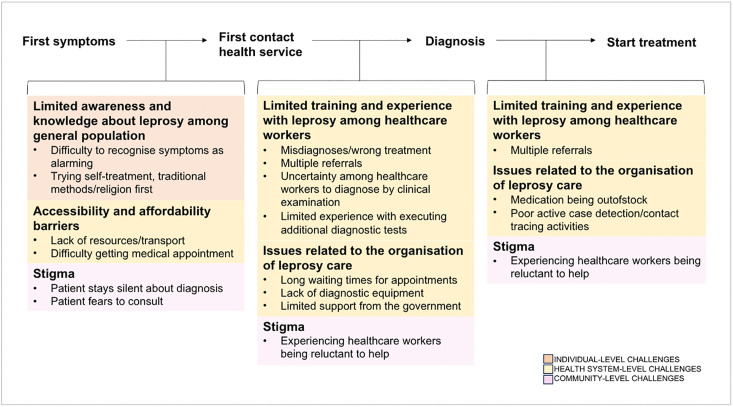
General patient pathway and reasons for diagnostic and treatment delay of leprosy according to people affected by leprosy in Cesar and Valle del Cauca, Colombia.

After exploring the diagnostic pathways with our participants, we inquired what they believed were reasons for delay in leprosy diagnosis in their own case or in broader context. Five major reasons were distinguished, including perceived limited training and experience with leprosy among healthcare workers, stigmatisation of people diagnosed with leprosy, challenges related to the organisation of leprosy care, accessibility and affordability barriers, and limited awareness and knowledge about leprosy among the general population. Below, we summarised the general patient route towards diagnosis and treatment, which shows the various challenges that may be causing delays in the different stages of that route (Fig 1). Although we summarised a generalised patient trajectory, it is important to highlight that each of our participants had their own unique experiences that did not often fit the linear understanding of diagnosis and treatment from the first symptoms to cure. Rather, some of the participants had multiple pauses in their diagnostic journeys as well as deviations from this trajectory following misdiagnosis. Therefore, while presented in a rather linear way on Fig 1, the diagnostic journey of leprosy is a dynamic and constantly changing process where patients had to enter and re-enter the different stages of this journey.

Below, we elaborate on the various individual-level, community-level, and health system-level challenges that were established along the patient trajectory.

#### Individual-level challenges.

To initiate the diagnostic journey, the first step is a visit to a healthcare worker. We distinguished two main challenges that our participants experienced to initiate this visit, including difficulty for the patient to recognise their symptoms as alarming and to find a right place within the healthcare system where they should go to. In the interviews, participants described that the first symptoms they noticed often were sensory loss or visible changes in skin, such as coloured skin patches. Due to loss of sensation, symptoms sometimes remained unnoticed by our participants. In some cases, due to symptoms occurring in places that are not immediately visible, such as a participant’s back, they had to rely on someone else to notice the symptoms. For example:

*“…one day I had taken a shower and got out in a towel, and my husband said to me: ‘Come here, love, you have a spot on your back.’ So I said to him: ‘A spot?’ He said: ‘Yes, a spot like a dark one.’ Then he said to me: ‘Shall I tell you where?’ He touched it, but well… I didn’t feel it there. And well… since we didn’t know about the disease, I told him: ‘But I don’t feel anything.’ So I started trying to touch it with my hand, but I didn’t feel anything.”* (person affected by leprosy diagnosed in 2014/2015, estimated delay of 4 years)

This shows how there can be a delay in the discovery of symptoms, specifically when they are located in less visible areas. Once noticing the symptoms, some participants did not recognise those as alarming and therefore did not seek medical help immediately. In some cases, months or even years passed by. Our participants indicated that they were not worried by their symptoms; they thought it was something temporary and sometimes skin symptoms seemed to indeed improve for a while. Further, as symptoms were often not painful, they did not trigger the need to seek immediate care, with some of the participants attributing their symptoms to other causes, such as work injuries or other accidents damaging the skin. For example:

*“When I was shaving my legs one time, and I noticed a rash on my leg, the right leg. So, I thought that it was… that it was just irritated due to shaving. I didn’t pay any attention to it.”* (person affected by leprosy diagnosed in 2021, estimated delay of 8-9 years)

Several of our research participants highlighted the general unfamiliarity with leprosy. They highlighted that it is not a commonly known disease and not something that they would generally encounter in media and public discussion. For instance, one participant explained:

*“…many people are unfamiliar with the topic [leprosy]. That’s why they also don’t worry about a skin patch that appears and how it progresses […]. In other words, people don’t worry that much that they would look at their body, do a check, look at a skin patch and see if.. if it changes in shape or colour and if they lose sensibility. So, that’s because they haven’t been given information about the disease.”* (person affected by leprosy diagnosed in 2022, estimated delay of 1 year)

And:

*“…there are campaigns for HIV, tuberculosis, and other diseases. And not Hansen’s disease. Not leprosy. […] Now that I start to think about it… I’ve never seen one [about leprosy], right? It’s never on television, I mean, why? […] It is being forgotten, […] Like I have always said that this disease is hidden, as if.. as if there is not much noise about it. That’s what we have always seen.”* (person affected by leprosy diagnosed in 2002, no delay)

When our participants did act upon their symptoms, some of them first used self-treatment or sought help in alternative ways before consulting the regular health system. This involved certain religious practices, using traditional methods, or applying homemade products or products from pharmacies. Using alcohol and natural products like ‘soap of the earth’ was mentioned. One participant consulted a medium to ask for help from José Gregorio Hernández, a physician from the nineteenth century who is believed to be responsible for various miracles. Unclarity of symptoms and treatment trajectories left people trying to find different cures for themselves, often leading to misdiagnosis, mistreatment and thus diagnostic delays. One of the participants experienced self-blame due to these experiences that left them with facial paralysis:

*“I never consulted and I think that that is what harmed me the most, because it gave me facial paralysis. And it affected my hands a lot.”* (person affected by leprosy diagnosed in 2021/2022, estimated delay of 1.5 years)

We distinguished various ways in which our participants eventually got in contact with the health services. First, this could occur by the participant’s own initiative. For instance, the following person explains how she started worrying when discovering that her mother was diagnosed with leprosy, after which she started seeking medical help herself:

*“…when they discovered my mom, that she already had those things [leprosy], I worried about my spot because it didn’t go away…”* (person affected by leprosy diagnosed in 2018/2019, estimated delay of 5-6 months)

Second, in some cases, people in the surroundings of the participant recommended or insisted that the participant would seek help. Usually, this person was worried about the participant or recognised the symptoms. In the third scenario, participants were visiting the health services for reasons other than leprosy-related, after which health staff was alarmed by certain symptoms of the patient. For example:

*“…I went to the dermatologist for the… for the League Against Cancer. She… yes, as soon as I took off my blouse, she says: ‘Mrs [], this isn’t mine, but I’m going to send you to where they are actually going to give you treatment that… that you need so that they cure what you have…”* (person affected by leprosy diagnosed in 2012, estimated delay of 1.5 years)

Lastly, one participant whose uncle was already diagnosed explained that a healthcare worker got in contact with her through active case detection, which was not the case for all of the participants who were in contact with active cases.

In summary, individual-level delay along the route towards diagnosis is embedded in overall difficulty to recognise leprosy symptoms and act upon it. Deeply rooted cultural habits, social arrangements and spiritual health beliefs may contribute to delayed or alternative health-seeking practices. Consequently, the individual-level delay suggests a structural lack of information provision and uptake about leprosy among the general population.

#### Community-level challenges.

Stigma is described by the participants as the belief that leprosy is highly contagious, deadly, or that you might be cursed when having leprosy, resulting in fear of being rejected or discriminated by fellow community members. Participants believed that it could prevent a patient from seeking help due to fear of rejection. Our findings highlight the occurrence of stigma in both community-related and health staff-related situations. In some cases, participants experienced reluctance from healthcare workers to help them once it became clear that the participant might have leprosy. For instance, the following participant was sent by her doctor to get an additional test in the hospital:

*“I went to where the nurses room was […] I asked a woman [nurse]: ‘look, they ordered this test.’ ‘What for?’ ‘Leprosy’ I said. And everyone looked… terrified. And I felt bad, of course, because you immediately feel something like rejection, like ‘Don’t get close to me.’ It’s ugly, but it still happens.”* (person affected by leprosy diagnosed in 2018/2019, estimated delay of 10 years)

This shows how courage, resilience and perseverance from the patient are necessary to withstand stigma-related situations from both the community and the health system itself. Therefore, our participants recommended implementing leprosy awareness and stigma-decreasing activities among both healthcare workers and the general population. Various strategies were mentioned, such as creating community groups that visit neighbourhoods and media campaigns. Additionally, the importance of involving people affected by leprosy in awareness activities was highlighted.

In summary, community-level delay is embedded in ongoing stigmatisation of people diagnosed with leprosy, in both community-related and health staff-related situations, therefore requiring community-wide action to tackle deeply rooted beliefs and misconceptions about the disease.

#### Health system-level challenges.

Health system-level challenges were a major cause for delay and were encountered in all stages of the patient pathway. In the first part of the pathway, we observed health system-level challenges in accessibility and affordability barriers impeding patients from timely contact with health services, such as a lack of financial resources or transport, or experiencing difficulty in getting an appointment. Further, logistical challenges in accessing healthcare can be seen between participants who live in urban and rural areas.

Further on in the pathway, delay may occur due to perceived limited leprosy knowledge and expertise among healthcare workers. Experiencing a route with multiple consultations, referrals, doctors, misdiagnoses and wrong treatments causing delays of months or years was a rule rather than an exception among our participants. To illustrate, one person stated:

*“Without lying or exaggerating, I went to more than 20 doctors and even specialists”* (person affected by leprosy diagnosed in 2012, estimated delay of 1.5 years)

This highlights the potential structural problem of limited training about leprosy symptoms and leprosy diagnosis among healthcare workers. According to our participants, leprosy symptoms were often confused with fungal dermatitis, allergies, or psychological causes by doctors. Other misdiagnoses included lupus, dust mites, parasites, melanoma, reaction to food, sun or medication, and sexually transmitted diseases. The lack of training among healthcare workers about leprosy as a potential cause for a patient’s symptoms was clarified by our participants’ stories in which the patient had to explain leprosy to their doctor, or in which the doctor started looking up the disease on the computer after it was mentioned by a patient. The following participant gave an example:

*“…they still tend to believe it’s a disease that no longer exists, […] I encountered doctors who are completely unaware of the disease, who tell me […] that it has already been eradicated. So they don’t consider it an option that those symptoms are from the disease [leprosy]. I mean, they don’t know it, they don’t know the symptoms. So, they rule it out completely and move on to other hypotheses that have nothing to do with it.”* (person affected by leprosy diagnosed in 2021, estimated delay of 8-9 years)

Our participants expressed feelings of frustration and disappointment, highlighting that they felt that their healthcare workers did not have enough expertise in diagnosing and treating leprosy. The following participant illustrates feelings of not getting an expert opinion from health staff who is supposed to give one:

*“…the reason is the doctor. There are many doctors who don’t have any experience. They are doctors because they have a degree. Because they like to be called doctor. But when it comes down to it, they have no knowledge, no experience.”* (person affected by leprosy diagnosed in 2012, estimated delay of 1.5 years)

Without finding a correct diagnosis, empathy from healthcare workers towards the patients was perceived by our participants as decreasing. For example, the following participant explained how she was hospitalised three times, potentially due to leprosy reactions, and wrongfully discharged:

*“So, he [doctor] told the nurse who took me to the appointment with him to please discharge me. That I had been occupying a bed that was actually needed for someone who was sick; that what I had was psychological, or something that I ate. So, they discharged me and took me out of the hospital.”* (person affected by leprosy diagnosed in 2014/2015, estimated delay of 4 years)

Our findings highlight that even when our participants gave ‘hints’ to healthcare workers, for example, by expressing their concerns about leprosy due to prior diagnosed family members, it either felt as if the doctor was not interested or the doctor reassured them that there was nothing to worry about. This emphasises how patients’ knowledge and expertise can sometimes be dismissed within the health system. The delays in diagnoses and dismissal of patients’ knowledge led some of our participants to disengage with the health system. Our participants described how they would lose interest in seeking help after multiple consultations without finding a correct diagnosis, and how such experiences would make them feel alone and isolated. For example:

*“I went to various dermatologists and they said that it was because I didn’t dry my skin properly, that it was a fungal infection, that… I don’t know what. I did a treatment for fungal infections. It [the symptoms] never went away. I left it alone and didn’t look at it again.”* (person affected by leprosy diagnosed in 2020, estimated delay of 6-12 months)

As a result, we observed that some participants decided to seek help elsewhere, for example by changing their Health Promoting Entity (EPS), which is responsible for providing access to certain health services:

*“It [skin patch] started to get bigger, it started to grow, grow, grow. […] And it spread a lot, to my legs, my back, my stomach, my arms, my body was completely covered with red patches. And they burned like crazy. […] I started to have difficulty gripping things. It hurt. I felt that I almost had no strength. […] And well, since they couldn’t find the solution, as they usually say, we decided to change EPS and start the search from scratch again. About eight years had already passed when I changed EPS.”* (person affected by leprosy diagnosed in 2021, estimated delay of 8-9 years)

One participant explained that she had to travel for approximately eight hours to visit doctors in a neighbouring department, revealing the challenging journeys that some participants have to take to find answers to their health problem. Our findings highlight that, besides misdiagnoses resulting in the progression of physical leprosy symptoms over time and ongoing transmission among the social contacts, misdiagnoses could also lead to a harmful psychosocial impact. For instance:

*“Approximately two or three years of that whole process of examination after examination passed. […] They even told me that it might be syphilis. […] They also ordered tests for sexually transmitted diseases for him [husband]. Obviously, our relationship was affected enormously because well… he started to distrust [me] a lot.”* (person affected by leprosy diagnosed in 2021, estimated delay of 8-9 years)

Our participants expressed that even when doctors did suspect leprosy and additional tests were ordered, a lack of medical expertise was shown during the execution of the tests or in the laboratory, which could eventually lead to erroneous test results. For example:

*“What a surprise it was when they did the additional test. They had no idea how to execute the test. […] They didn’t know. There were many interns and since it was the first time that they did this test, it was kind of new. So the nurse was teaching the interns how to do it even though she didn’t know it either. So, they were reading a book to see how to do it. And learning with me.”* (person affected by leprosy diagnosed in 2021, estimated delay of 8-9 years)

When considering the moment of diagnosis, nearly all participants described that their diagnosis was established due to additional tests instead of clinical examination, suggesting the limited expertise among healthcare workers to diagnose clinically. In some cases, participants did not receive an explanation about the results from the healthcare worker who performed the additional test. Therefore, they had to search the internet themselves or were referred again to another doctor to receive their diagnosis. For example:

*“The skin doctor [dermatologist] over there performed the biopsy and gave me the results. However, since one didn’t know what Hansen’s disease was, I had to make an appointment again with the general practitioner and she told me that it was leprosy.”* (person affected by leprosy diagnosed in 1992, estimated delay of 6 months)

And:

*“When my husband went back to receive the results, since I was so ill, and he told me: ‘here love, look, the results’. And I looked at it and said, Hansen […] I had never heard about that. So, you… it’s like a human thing, the first thing you do is go to Google. And when it said that it was leprosy, and there were very disturbing photos of people that didn’t have their nose, lost their eyes, their feet were eaten away, I started to cry and told my husband: ‘what?’ Well, I believed that I was the only person on Earth that had this disease, and I cried for three days because I said to my husband that I was going to die. And be without arms, or without a nose, without many parts of my body.”* (person affected by leprosy diagnosed in 2014/2015, estimated delay of 4 years)

This highlights how certain misconceptions about leprosy could be strengthened without sufficient guidance during the diagnostic process and psychosocial support for the patients. Other patients highlighted that they believed that their diagnosis was related to God or being surprised; participants mentioned that they never expected it to be leprosy or that they were not familiar with the disease. Additionally, some participants felt frightened because they thought that they were going to die, be abandoned by their community, or sent to a leprosarium. Hence, some decided to keep their diagnosis silent, which shows the established stigmatising attitudes towards leprosy. For example:

*“…I am going to tell you something. There is a part of my family that doesn’t know that I suffered from this disease. […] And they are not going to know it, definitely not.”* (person affected by leprosy diagnosed in 2018/2019, estimated delay of 10 years)

Other health system-level reasons for delay are related to challenges with the organisation of leprosy care, ranging from experiencing difficulties in getting an appointment to medication being out of stock. The lack of continuity of health staff, for example by short-term temporary contracts, is believed by our participants to complicate successful implementation of the leprosy program, specifically active case detection and contact tracing activities. As a recommendation to lower diagnostic delays, our participants emphasised to properly execute active case detection and contact tracing activities, specifically in remote areas and among families of persons diagnosed with leprosy. Additionally, participants suggested prioritising leprosy diagnostic tests for patients with skin symptoms, indicating improvements in diagnostic protocols for dermatological patients. Also, equipment availability issues were described:

*“…just because they didn’t have a forceps, I had to wait like three months for the slit skin smear test.”* (person affected by leprosy diagnosed in 2016, estimated delay of 3 months)

These events lead to participants sensing limited support from the government. A call was made to strengthen overall commitment towards leprosy-related activities and more understanding for leprosy patients to ensure that patients do not feel as if they are ‘orphans’ or ‘dying without dying’ during their search for help. The following participant emphasised this:

*“…the recommendation is that everyone, whether doctor, patient or public health official, should not give up and should not allow this disease to continue, continue as if forgotten, still pretending and believing that it is a disease from the Bible”* (person affected by leprosy diagnosed in 2019, estimated delay of 2 years)

In contrast, several participants highlighted their positive experiences with the Pan American Health Centre (Cali, Valle del Cauca). Once they were referred there, participants felt that their diagnosis and treatment were quickly organised.

In summary, various challenges are encountered within the health system resulting in both diagnostic and treatment delays. A key element that seems to underlie many of those challenges involves perceived limited training among healthcare workers regarding leprosy symptoms, which may be related to the limited experiences healthcare workers have with leprosy and its low priority within the national health system. Hence, our participants emphasised that doctors - specifically younger doctors and medical students - nurses and health officials/management staff should be taught about the existence of leprosy, the importance of a timely diagnosis and trained in abilities to diagnose clinically. Giving time-to-time lectures or handing out flyers at universities and hospitals were mentioned as potential strategies.

## Discussion

Our study illustrates the complexity of reasoning behind the diagnostic delay of leprosy and highlights the impact this had on people affected by leprosy in the two endemic departments Cesar and Valle del Cauca in Colombia. Delay often consists of the interplay between individual-level, community-level, and health system-level challenges, frequently resulting in an accumulation of delay time spanning several years in our study population. The multitude of challenges eventuates in near impossibility for leprosy patients to avoid at least some degree of delay and requires strong perseverance from the patient to find a correct diagnosis. Following the experiences of people affected by leprosy, our findings highlight that leprosy symptoms may be difficult to recognise by both patients and healthcare workers. The limited medical leprosy expertise and overall awareness among the public could suggest a structural problem of lacking infrastructure that guides the public towards more effective health-seeking practices and implies that current national and regional leprosy-related activities seem to provide insufficient support to ensure a timely diagnosis. While robust surveillance systems have been marked as a key component to eliminate leprosy, the findings of our current study as well as our previous research among leprosy health professionals highlight shortcomings in systematic active case detection and contact tracing activities [1,11]. We can suggest that due to a low incidence of leprosy cases in Colombia, the disease was deprioritised on the national health agenda [25], a trend that has been observed in other low-endemic countries [1,26,27].

The findings of our study are in line with previous regional research on this topic. Our study confirms the suggestion by Barbosa Ladino and colleagues that the diagnosis often seems to depend on ‘luck’, for example, by encountering a doctor who happens to recognise the symptoms [10]. The various reasons for delay are consistent with the results of our previous research in Cesar and Valle del Cauca which included leprosy health professionals. However, whereas referral and authorisation procedures within the complex organisation of the Colombian health system have been mentioned as a cause for delay in the previous research, this was not emphasised as a major reason for delay by people affected by leprosy in our current study [11]. Our findings are also in line with similar research from other continents, except for the role of gender not being mentioned as a reason for delay in our study [3,28].

### Limitations

This study holds particular significance given the endemic nature of leprosy in the researched area. Nevertheless, it is important to acknowledge several limitations of the study. First, only two endemic departments were included, meaning that the results are not generalisable to the whole of Colombia. Second, regarding the study population, our study did not include children in the study population, while lowering diagnostic delay among children is crucial for disability prevention. In addition, using the GLRA/DAHW network for convenience sampling might have introduced a sampling bias and social desirability bias due to the participants’ connection to GLRA/DAHW. Due to sampling from patient meetings, our study population might over represent particularly engaged people affected by leprosy relative to the general population of those affected by leprosy in Colombia. Third, our research focused on the experiences and perspectives of people affected by leprosy, which calls upon the participants’ memories. The possibility of some degree of recall bias could therefore not be ruled out. Along these lines, it is worth emphasising that our research highlights the experiences of only people affected by leprosy, meaning that conclusions are mainly based on their experiences and perceptions. This makes our study a valuable addition to our previous regional research that investigated the perceptions by leprosy health professionals with diagnostic delay [11].

### Implications

Our findings might contribute to leprosy policy decision-making in the study setting. To fulfil the objectives of the Colombian Ministry of Health and Social Protection and the WHO to eliminate leprosy, structural improvements in the leprosy care pathway seem pivotal. While national and international guidelines highlight clinical examination as the cornerstone for diagnosis, our findings suggest that healthcare workers currently possess limited expertise to correctly diagnose via clinical examination. This reflects the discrepancy between theoretical intentions and daily healthcare realities.

Regarding leprosy control activities, the question of centralising or decentralising leprosy care has been a point of discussion before [29]. This highlights the dilemma between prioritising specialised expertise (centralised care) versus prioritising accessibility (decentralised care), while also taking cost-effectiveness and feasibility into account. Arguments can be suggested for both, but highly depend on study settings. Whereas in high-endemic settings decentralising care might be the common strategy, as argued by Van Brakel and colleagues, the context of our study shows a lower endemic setting with 200–300 new leprosy cases annually [29]. Considering the low prevalence of leprosy and limited exposure of healthcare workers to leprosy patients, it is desirable to apply a dual approach that on the one hand centralises leprosy care in appointed centres that are responsible for integral leprosy care and experienced in diagnosing both clinically and by additional tests [29]. The positive experiences of people affected by leprosy with the Pan American Health Centre (Valle del Cauca) support this. On the other hand, healthcare workers should be trained about the existence, symptoms and importance of a timely diagnosis of leprosy. A roadmap showing healthcare workers where to refer to when they suspect a patient of leprosy is crucial for efficient referrals and to bypass delays. To ensure the effective functioning of integral leprosy centres, commitment is necessary on a national and local level in terms of the provision of financial and human resources, and permanent employment contracts.

Furthermore, our findings imply prioritising active case detection and contact tracing activities. Even though various participants in our study had family members who were already diagnosed with leprosy, only one participant was diagnosed via active case detection. This suggests the shortcomings of current protocols for contact tracing activities. The participant who was found via active case detection suffered no delay, which suggests the positive effects of contact tracing activities on delay. It is highly recommended to improve protocols to ensure consistent follow-through of active case detection. Besides the need for structural improvements within the leprosy care pathway, our study also highlighted ongoing stigmatisation of the people diagnosed with leprosy in both community- and health system-related situations. The implementation of awareness strategies among the general public and healthcare workers is therefore pivotal to tackle ongoing misperceptions about the disease and promote more effective health-seeking practices. Based on the results of this study and following the suggestions of our research participants, we summarised the main recommendations to diminish diagnostic delays of leprosy in Cesar and Valle del Cauca (Table 3).

**Table 3:** Recommendations to lower diagnostic delays in Cesar and Valle del Cauca based on our study.

1. Prioritise active case detection and systematic contact tracing
2. Appoint/improve integral leprosy centres that are responsible for diagnosis, treatment, follow-up, active case detection and contact tracing activities
3. Implement strategies to raise awareness and build expertise among healthcare workers (and medical students) about the existence, symptoms and importance of a timely diagnosis of leprosy • Emphasise including leprosy in the differential diagnosis of patients with skin symptoms and/or sensory loss
4. Implement and promote a roadmap for healthcare workers that shows what to do and where to refer to when suspecting leprosy
5. Implement strategies to decrease stigma in community-related and health system-related situations
6. Implement awareness activities targeting the general population to improve symptom recognition. For example, via campaigns on radio/television or social media • Specific focus on reaching vulnerable groups (e.g., lower socioeconomic status or rural residence)

It is recommended to include people affected by leprosy in awareness activities and active case detection programs. This could positively contribute to creating awareness about the impact and consequences of delay.

In conclusion, our study maps the patient pathway towards diagnosis and highlights the underlying reasoning behind diagnostic delay in Cesar and Valle del Cauca, following the experiences and perceptions by people affected by leprosy. Diagnostic delay is complex and often an accumulation of multiple individual-level, health system-level, and community-level challenges, resulting in a negative physical and psychological impact on the patient. Therefore, it requires a multifactorial approach to lower diagnostic delays in our study setting.

## Supporting information

S1 Appendix Interview guide (English version). (DOCX)
