# Supplementary material for: Experiences and perceptions on diagnostic delay of leprosy by affected people in Colombia: A qualitative study
Source: PLoS Negl Trop Dis. 2026 Jun 11;20(6):e0014412. doi: 10.1371/journal.pntd.0014412 (PMC13258010; doi:10.1371/journal.pntd.0014412)
Supplement: S1 Appendix — (DOCX) [file pntd.0014412.s001.docx]

**S1: Interview guide (English version)**

| INTRODUCTION |
| --- |
| - Interviewer introduces him/herself and the study. - Interviewer gives instructions about the interview. - Interviewer asks if participant has any questions about the interview/study. - (Interviewer is free to discuss any other topics/small talk to create trust.) - **Informed consent:** if written informed consent was not possible, verbal consent needs to be obtained and recorded. |

| SEMI-STRUCTURED QUESTIONS | |
| --- | --- |
| Obtaining background data from the participant  Exploring knowledge about ‘Leprosy’ and type: paucibacillary/  multibacillary | 1. **Could you tell me about yourself? It is not necessary to mention your name.**  - **How old are you?** - **Where do you live? (city? rural?)** - **Do you identify as man, woman or other?** - **Do you work?** (if yes) **What kind of work do you do?** - **Did you receive any formal education?** (if yes) **Where did you receive your education from?**  1. **Could you tell me about the disease you are diagnosed with?**  - **Do you know what it is called?** (If the participant uses another word to describe Leprosy disease, the interviewer will use this same word to refer to Leprosy disease throughout the interview). - **Since how long have you been taking treatment?** - **How long did the doctor mention the treatment will take?** |
| Exploring route to diagnosis | 1. **In your own words, could you briefly guide me through your journey to getting diagnosed?**  - How and when did you notice the first symptoms? What did you think? (Were you thinking about Leprosy?) What did you do? Why? - When did you visit a doctor, why? What happened afterwards? What do you think about the consult with the doctor(s)? - How much time did pass between noticing your first symptoms and the moment of diagnosis? |
| Exploring the reasons for delay in diagnosis | 1. **From previous studies, we know that some time can pass before someone receives a diagnosis. There might pass weeks, months or years between the moment that someone notices the first symptoms and the moment of obtaining the diagnosis.** For example, you told me… **This time is what we call the ‘delay’.**   **In your opinion, what are the reasons for this delay in diagnosis?**   - What are the reasons for not immediately receiving Leprosy diagnosis after noticing the first symptoms? - Reasons related to people (e.g family, community, health worker…) - Reasons related to setting (e.g. access to health system, resources/money, stigma, materials…)  1. **Which barriers did you experience to get Leprosy diagnosis?**  - In your opinion, which reasons can you think of that caused you to not receive Leprosy diagnosis immediately after noticing the first symptoms?  1. **Which barriers do you think others experience to get Leprosy diagnosis?** 2. **A previous study conducted in Colombia** (Gómez et al., 2018) **showed that the majority of Leprosy patients do not seek help after noticing the first symptoms.**   **What do you think about this?**   - Do you think this is true/false? Why? (if yes) What do you think are the reasons that people do not seek help immediately?  1. **In that same study, it was found that 27% of Leprosy patients need five or more consultations before they receive the Leprosy diagnosis.**   **What do you think about this?**   - Do you think this is true/false? Why? (if yes) What do you think are the reasons that patients need five or more consultations before receiving Leprosy diagnosis?  1. **Before moving on, are there any other reasons for delay in diagnosis that we have not discussed yet?** |
| Exploring suggestions for improvement | 1. **In your opinion, what do you think is necessary to receive Leprosy diagnosis more quickly? Why do you think so?** |

| ENDING |
| --- |
| - These were my questions. Is there anything else related to the topic that we have not covered yet and that you would like to discuss? - How do you feel about the interview? Do you have any questions? - Thank you very much for your participation. May we contact you again in case additional questions come up? - Would you like to receive the results of the study once it is finished? (if yes) How can we reach out to you? |
